# Editorial: Brain connectomics: a comprehensive mapping and analysis of brain connectivity in health and disease

**DOI:** 10.3389/fmed.2026.1900109

**Published:** 2026-06-16

**Authors:** Mario Petretta, Giorgio Treglia, Alberto Cuocolo

**Affiliations:** 1 Department of Advanced Biomedical Sciences, University of Naples Federico II, Naples, Italy; 2 Division of Nuclear Medicine, Imaging Institute of Southern Switzerland, Ente Ospedaliero Cantonale, Lugano, Switzerland; 3 Faculty of Biomedical Sciences, Università della Svizzera Italiana, Lugano, Switzerland; 4 Faculty of Biology and Medicine, University of Lausanne, Lausanne, Switzerland; 5 IRCCS Synlab SDN, Naples, Italy

**Keywords:** brain network, deep learning, neuroimaging, persistent homology, structural covariance network

Recent methodological and clinical studies have significantly advanced our understanding of brain connectivity patterns in both health and disease through innovative topological and neuroimaging analyses. We are pleased to present the Research Topic “*Brain connectomics: a comprehensive mapping and analysis of brain connectivity in health and disease*.” We extend our gratitude to everyone who contributed to this Research Topic, especially the researchers who responded to our invitation and the reviewers whose valuable comments greatly enhanced its quality.

The terms “connectome” and “connectomics” have become popular since Sebastian Seung's work in 2012 (1). Mapping the connectome allows to see how signals travel through the brain, revealing the information highways that harmonize our daily actions. Experts engaged in this important field of research wrote the articles in this topic. These works cover theoretical, methodological, and practical advances in areas of clinical interest. This presentation hopes to encourage readers to delve deeper into the individual contributions.

Recent methodological and clinical studies have significantly advanced our understanding of brain connectivity patterns in both health and disease through innovative topological and neuroimaging analyses. Pioneering network-level metrics, Garai et al. introduced H-VIP (Homological Vertex Importance Profile), a novel metric rooted in persistent homology that quantifies the contribution of individual brain regions to global connectivity and cyclic organization across spatial scales. Validated on independent cohorts from the Human Connectome Project (HCP) and the Alzheimer's Disease Neuroimaging Initiative (ADNI), H-VIP outperformed traditional degree and betweenness centrality metrics, demonstrating great potential for mapping topological changes linked to neurodegenerative progression.

The clinical translation of advanced connectivity analyses is further exemplified in developmental and pain disorders. Investigating neurodevelopmental interventions, Martinez Agulleiro et al. conducted a clinical trial involving children with organizational skills deficits to evaluate a 10-week online Behavioral Organizational Skills Training (OST). Utilizing resting-state functional magnetic resonance (MR) imaging across three analytical workflows, they assessed functional connectivity between the dorsal anterior cingulate cortex and the anterior striatum within the default mode, frontoparietal, and limbic networks. While parent and teacher feedback confirmed the behavioral benefits of the online intervention, associated microstructural changes in brain connectivity remained uncertain, highlighting the need for further investigation.

Conversely, Liu et al. leveraged structural network analysis to monitor patients with chronic neck and shoulder pain (CNSP) before and 3 months after a minimally invasive intervention. They reported widespread post-treatment reductions in cortical thickness, most notably within the posterior cingulate cortex, alongside decreases in baseline degree centrality and nodal efficiency within the right pars triangularis. Crucially, the intervention led to a significant recovery of nodal connectivity in the pars triangularis, and its baseline centrality level successfully predicted the magnitude of pain relief (*r* = −0.47, *P* = 0.0168), underscoring the value of structural biomarkers for personalized medicine.

Advanced neuroimaging has also shed light on the complex neurobiology of sleep and cognitive preservation. Jin et al. employed resting-state functional MR imaging and morphometric analyses to investigate brain alterations in primary insomnia, a condition heavily tied to cognitive decline and metabolic risks. Their findings revealed reduced functional connectivity between the central executive network and both the right cerebellum and posterior default mode network, with the precuneus and posterior cingulate acting as aberrant hubs that support typical hyperarousal patterns. Intriguingly, while widespread tissue thinning and decreased fractal dimensions pointed to sleep-fragmentation wear, a focal increase in frontoparietal gyrification was observed (2). This morphological discrepancy reflects an independent regional compensatory effort, suggesting that local geometric complexity is maintained via beneficial neuroplasticity to preserve wiring efficiency and mitigate daytime functional deficits (3).

Finally, to bridge the gap between complex network metrics and clinical utility, Xu et al. developed GenCPM, an open-source R toolbox that integrates traditional Connectome-based Predictive Modeling (CPM) with Generalized Linear Models (GLMs). Validated using real-world data from major cohorts such as Alzheimer's Disease Neuroimaging Initiative (ADNI) and the Anti-Amyloid Treatment in Asymptomatic Alzheimer's (A4) study, GenCPM significantly outperformed traditional frameworks in predicting cognitive decline and pathological staging. By transforming complex functional MR/diffusion MR scans into quantifiable biomarkers, natively accounting for demographic covariates (such as age and education), and supporting binary or count data, this toolbox enables clinicians to accurately stratify pre-symptomatic patients, moving personalized risk scoring closer to practical medical application.

Taken together, these studies underscore a clear trajectory in neuroimaging research toward highly translational, network-centric methodologies. Whether exploring mathematical frameworks like persistent homology, monitoring treatment-induced plasticity in chronic conditions, or capturing compensatory structural alterations in the sleep-deprived brain, the literature highlights the critical role of topology in decoding brain function and pathology. Moreover, the emergence of open-source predictive frameworks like GenCPM represents a crucial step toward clinical implementation. Ultimately, shifting the focus from group-level descriptions to individualized, connectome-based biomarkers hold the key to uncovering the subtle mechanisms of neurodegeneration and delivering truly personalized diagnostic and therapeutic strategies.
